# Application of Endobronchial Ultrasonography for the Preoperative Detecting Recurrent Laryngeal Nerve Lymph Node Metastasis of Esophageal Cancer

**DOI:** 10.1371/journal.pone.0137400

**Published:** 2015-09-15

**Authors:** Hong-Bo Shan, Rong Zhang, Yin Li, Xiao-Yan Gao, Shi-Yong Lin, Guang-Yu Luo, Jian-Jun Li, Guo-Liang Xu

**Affiliations:** 1 Department of Endoscopy, Sun Yat-sen University Cancer Center, Guangzhou, China; 2 State Key Laboratory of Oncology in South China and Collaborative Innovation Center for Cancer Medicine, Sun Yat-sen University Cancer Center, Guangzhou, China; Baylor College of Medicine, UNITED STATES

## Abstract

**Background:**

The preoperative detection of recurrent laryngeal nerve lymph node (RLN LN) metastasis provides important information for the treatment of esophageal cancer. We investigated the possibility of applying endobronchial ultrasonography (EBUS) with conventional preoperative endoscopic ultrasonography (EUS) and computerized tomography (CT) examination to evaluate RLN LN metastasis in patients with esophageal cancer.

**Methods:**

A total of 115 patients with advanced thoracic esophageal cancer underwent EBUS examinations. Patients also underwent EUS and CT imaging as reference diagnostic methods. Positron emission tomography /computed tomography (PET/CT) was also introduced in partial patients as reference method. The preoperative evaluation of RLN LN metastasis was compared with the surgical and pathological staging in 94 patients who underwent radical surgery.

**Results:**

The sensitivities of the preoperative evaluations of RLN LN metastasis by EBUS, EUS and CT were 67.6%, 32.4% and 29.4%, respectively. The sensitivity of EBUS was significantly different from that of EUS or CT, especially in the detection of right RLN LNs. In addition, according to the extra data from reference method, PET/CT was not superior to EBUS or EUS in detecting RLN LN metastasis. Among all 115 patients, 21 patients who were diagnosed with tracheal invasions by EUS or EBUS avoided radical surgery. Another 94 patients who were diagnosed as negative for tracheobronchial tree invasion by EUS and EBUS had no positive findings in radical surgery.

**Conclusions:**

EBUS can enhance the preoperative sensitivity of the detection of RLN LN metastasis in cases of thoracic esophageal cancer and is a useful complementary examination to conventional preoperative EUS and CT, which can alert thoracic surgeons to the possibility of a greater range of preoperative lymph node dissection. EBUS may also indicate tracheal invasion in cases of esophageal stricture.

## Introduction

Because most clinical manifestations of esophageal cancer occur in the advanced stages, surgery is the primary clinical treatment for the management of patients. Proper preoperative evaluations of cancer invasion and loco-regional lymph node metastasis are fundamental in therapeutic planning for esophageal cancer treatment. Currently, esophageal cancer staging is commonly performed by endoscopic ultrasonography (EUS) and computerized tomography (CT). EUS has advantages in the determination of peritumoral lymph node metastasis and the depth of cancer invasion (T stage), whereas CT provides a more helpful assessment of distant lymph node metastasis and metastasis to other organs [1]. Recurrent laryngeal nerve (RLN) lymph node metastasis provides important staging information for the treatment of esophageal cancer. The additional removal of RLN lymph nodes may improve the 5-year overall survival and disease-free survival rates of patients with esophageal cancer [2]. The status of RLN lymph node metastasis is considered an indicator of supraclavicular lymph node dissection in patients with advanced esophageal cancer. Even in cases of unilateral RLN lymph node metastasis, bilateral supraclavicular lymph node dissection should be recommended [3]. Furthermore, recurrent nerve node metastasis is a possible risk factor for cervical node metastasis [4]. However, in clinical practice, we found that ultrasounds can be attenuated by air that is present inside the trachea and bronchi and that this limits the use of EUS in the detection of metastases in the para-esophageal lymph nodes, especially in instances of RLN lymph node metastases that are close to the trachea. In recent years, endobronchial ultrasonography (EBUS) has become complementary to mediastinoscopy and is important in the diagnosis of mediastinal lymph node metastases in lung cancer [5,6]. Therefore, EBUS is a potential tool for the diagnosis of RLN lymph node metastases in patients with thoracic esophageal cancer because the position of the ultrasonic probe placed at the tip of the bronchoscope allows for the visualization of the RLN lymph node without the interference of air. Moreover, many patients with advanced stage esophageal cancer have esophageal stricture. Some of these patients cannot undergo EUS in preoperative evaluations because the echoendoscope cannot pass through the stricture. EBUS also permits the diagnosis of RLN lymph node metastases and tracheobronchial tree invasion by esophageal cancer from the transbronchial side.

In this study, we performed preoperative evaluations with both EBUS and EUS for the diagnosis of patients with esophageal cancer who were in an advanced stage, and we investigated the possibility of the application of EBUS with conventional preoperative EUS and CT examination for the evaluation of RLN lymph node metastases in patients with thoracic esophageal cancer.

## Patients and Methods

### Patient selection

#### Inclusion criteria

Patients were first diagnosed with thoracic esophageal cancer by a barium meal examination and gastroscopic biopsy. Criteria also included the absence of severe diseases of the heart, lungs, liver, kidney, and other major organs as well as neoplasms that were resectable with curative surgery.

#### Exclusion criteria

The exclusion criteria included patients with severe cardiopulmonary dysfunction or other severe diseases of the major organs (e.g., liver and kidney); tumors that were unresectable due to, e.g., distant metastases found by preoperative CT examination, tracheal invasions revealed by conventional bronchoscopy, and aortic invasions discovered by CT examination or EUS; patients who refused EBUS and EUS; patients who had received chemotherapy and/or radiotherapy before the examinations; and cases where inadequate information was collected such that a statistical analysis could not be conducted.

The study was conducted in accordance with the Declaration of Helsinki and was approved by the Ethics Committee of our institution. Patients were informed of the investigational nature of the study and provided their written informed consent.

In this study, we performed all tests in a blinded manner. The endoscopists who performed EBUS and EUS as well as the pathologists who handled the patient’s samples did so independently and provided a diagnosis for each patient based on his or her own observations.

#### Characteristics of the patients

A total of 115 patients (age range, 44–82 years; average age, 58.9±7.9 years; 85 males and 30 females), were recruited for this study from June 2013 to June 2014. All patients were diagnosed with esophageal squamous cell carcinoma by pathologists.

### EUS examination

The EUS used in this study was the Olympus EU2000 (Tokyo, Japan) radial endoscopic ultrasound system, and the conventional gastroscope used was the Olympus GIF240 (Tokyo, Japan). Patients fasted for 4–6 hours before the EUS examination, and 10 ml of 1% dyclonine was administered orally for topical anesthesia; anisodamine 10mg was administered by intramuscular injection 10–15minutes before the examination to reduce nausea and gastrointestinal peristalsis. Patients were placed in the left lateral position for this procedure. Prior to EUS, a conventional gastroscopy was performed to determine the location of the malignancy and the severity of the esophageal stricture. Then, EUS was performed with a scanning frequency of 7.5MHz; withdrawal from the descending part of duodenum was followed by examination of the stomach and the esophagus. In the stomach, we used the luminal water-filling method, whereas in the esophagus, we used the water-filled balloon combined with the luminal water-filling technique. Each patient was examined sequentially for celiac lymph nodes, gastric/esophageal regional lymph nodes, and the invasion depths of the esophageal neoplasms.

The location of the lesions was based on the distance of the ultrasonic probe to the central incisor and on the adjacent anatomic location, such as the celiac artery, crus of the diaphragm, left inferior pulmonary vein, azygos arch, aortic arch, tracheal bifurcation, and thyroid, among others. The invasion depths of the primary lesion of the esophageal cancer were assessed. The size and location of metastatic lymph nodes were also recorded.

### EBUS examination

The EBUS used in this study was the Olympus BF-UC260F-OL8 (Tokyo, Japan) with a convex probe, and the conventional bronchoscope used was the Olympus BF-240. Patients fasted for 4–6hours before the EBUS examination. Patients were intravenously injected with 2 mg of midazolam 5 minutes before the examination for sedation, and this was occasionally supplemented with another 2 to 3mg administered during the procedure. First, a conventional bronchoscope was inserted through the nose, and we sprayed a 2% lidocaine saline solution onto the mucosal surface for anesthesia of the airway using the conventional bronchoscope as a guide. Then, an ultrasound bronchoscope was inserted through the nose (or mouth if the patients had a narrow nasal cavity) to examine the peribronchial metastatic lymph nodes; we also investigated potential invasion of the tracheobronchial tree by the esophageal cancer. EBUS was conducted by inserting an ultrasound probe that was placed at the tip of the bronchoscope with a water-filled balloon in direct contact with the tracheobronchial wall. The scanning frequency was 7.5MHz. Regional lymph nodes were classified by the Mountain classification method [6]. To detect the right RLN lymph nodes, maintain contact with the trachea and withdraw the bronchoscope further to visualize the bifurcation of the right brachiocephalic artery, the tip was turned clockwise to visualize the esophagus on the ultrasound image, and then the right carotid artery was followed until the upper borders of the thyroid gland. The lymph nodes above the bifurcation of the right brachiocephalic artery and between the right carotid artery and esophagus are the right RLN lymph nodes. To detect the left RLN lymph nodes, the tip was turned counterclockwise at the main carina to visualize the aortic arch, and then the left carotid artery was followed until the upper borders of thyroid gland. The lymph nodes above the lower edge of the aortic arch and between the left carotid artery and esophagus are the left RLN lymph nodes. The relationship between the trachea or the left main bronchus with the esophageal cancer was determined by ultrasonic scan, and the location and size of the paratracheal and peribronchial lymph nodes were recorded.

### Diagnostic criteria of lymph node metastasis by morphologic features

By EBUS and EUS, the metastatic lymph nodes were diagnosed based on 1) a round shape (the ratio of the long to short axis of lymph nodes was <1.5 with curved border), 2) a large size (a maximum axis diameter ≥10mm, plus the ratio of short axis/long axis >1/2), 3) a distinct margin, 4) echogenic features, i.e., the presence of a hypoechoic or heterogeneous internal echo, and 5) an obliterated hilar architecture. If a lymph node did not meet all of the criteria, the imaging score was used as the determinant [7]. The metastatic lymph nodes were diagnosed and their location was recorded.

As a reference diagnostic method, diagnostic CT imaging for esophageal regional lymph node metastasis was performed according to the maximum axis diameter ≥10mm and marginal or partial lymph node reinforcement [8]. 18F-fluorodeoxyglucose (18F-FDG) positron emission tomography /computed tomography (PET/CT) was also introduced in partial patients as reference method. Metastatic lymph node was definite only when the radionuclide distributed uniformly and edge clearly under the precondition of standard uptake value (SUV) >2.5. The metastatic lymph nodes were diagnosed and their location was recorded.

### Surgical and pathological staging

Cases of esophageal cancer were staged according to the depth of cancer invasion and lymph node metastasis, which were noted in the postoperative pathology report and were observed during the radical surgery and radical regional lymph node dissection, including the lymphadenectomy of RLN lymph nodes, combined with the postoperative pathological report.

### Statistical analysis

Statistical analyses were performed using the SPSS 17.0 program. The morphologic features of EBUS were analyzed by logistic regression analysis. The clinical value of the EUS, EBUS, and CT methodologies included sensitivity, specificity, positive predictive value and negative predictive value. Sensitivity was analyzed by the McNemar Test. *P* <0.05 was considered statistically significant.

## Results

A total of 115 patients (age range, 44–82 years; average age, 58.9±7.9 years; 85 males and 30 females) were recruited for this study from June 2013 to June 2014 (see Table 1). All patients were diagnosed with esophageal squamous cell carcinoma by pathologists. This included 4 tumors located in the upper thoracic esophagus, 78 located in the middle thoracic esophagus, and 33 located in the lower thoracic esophagus.

**Table 1:** General information about of patients.

			N = 115
Male/female			85/30
Age, median(range), years			
59 (44–82)			
Tumor Location			
	upper thoracic esophagus		4
	middle thoracic esophagus		78
	lower thoracic esophagus		33
Treatment			
	radical surgery		94
	radiotherapy and chemotherapy		21
Patients with RLN lymph nodes metastasis			
	right		22
	left		7
	both sides		6

The general information about the patients is presented in Table 1. Among all of the patients, 21 patients who were diagnosed with T4 stage by EUS or EBUS did not undergo radical surgery but instead received radiotherapy and chemotherapy. Among these 21 patients, 14 patients were shown to have tracheal invasions by EBUS examination. Nine of these 14 patients failed to be assessed by EUS due to obstructive esophageal tumors. Another 7 patients were diagnosed with tracheal invasions by both EUS and EBUS. Overall, 94 of the 115 patients were treated with radical surgery, and no cancer invasion to adjacent organs (T4 stage) was found in any of these patients.

A total of 573 RLN lymph nodes were dissected in this study, and 69 RLN lymph nodes of 35 patients were shown to have metastases by postoperative pathology. Of these, 22 patients had metastases in the right RLN lymph nodes, 7 patients had left RLN lymph node metastases, and 6 patients had RLN lymph nodes metastases at both sides.

In this study, 3 patients were highly suspected to have para-RLN lymph node metastases, therefore, they received endobronchial ultrasound-guided transbronchial needle aspiration (EBUS-TBNA). The results of the fine needle aspiration biopsy found lymph node metastases.

**Fig 1:**
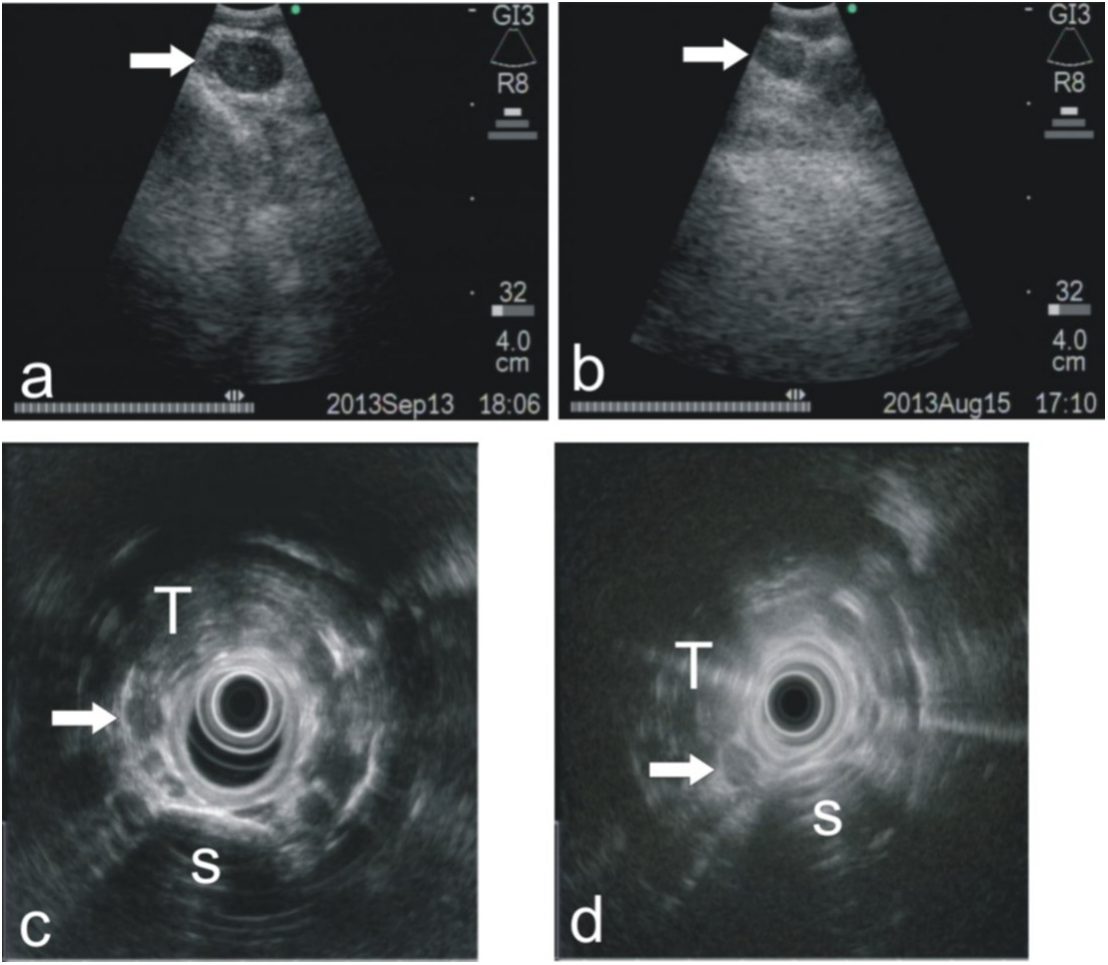
Representative images for EBUS and EUS in detecting RLN lymph node metastases. **(**a) An EBUS image showed an enlarged right RLN lymph node with a round shape, a distinct margin and a hypoechoic internal echo with obliterated hilar architecture. The lymph node metastases was diagnosed by EBUS. The patient accepted radical resection of the esophageal carcinoma. The postoperative pathology proved right RLN lymph node metastases. (b) An EBUS image indicated a benign right RLN lymph node with an iso-echoic internal echo and indistinct margin and non-round shape. The postoperative pathology showed a negative result at the right RLN lymph node group. (c) An EUS image indicated a metastatic right RLN lymph node with a round shape and a hypoechoic internal echo with obliterated hilar architecture. The postoperative pathology proved right RLN lymph node metastases. (d) An EUS image indicated a benign right RLN lymph node with an iso-echoic internal echo and non-round shape. The postoperative pathology showed a negative result for the right RLN lymph node group. T, trachea; S, spine.

Some representative images for EUS and EBUS in the detection of RLN lymph node metastases are shown in Fig 1. The morphologic features of EBUS were analyzed by logistic regression analysis. As shown in Table 2, a round shape and margins were independent predictive factors (*P* <0.05), with respective odds ratios of 4.56 and 0.13, respectively. A round shape was the most influential morphologic predictor of RLN lymph node metastases.

**Table 2:** Logistic regression analysis of EBUS image categories for the prediction of RLN lymph node metastases compared surgical pathology.

Morphologic Category	odds ratio(OR)	95% CI for OR	P Value
Size	2.02	0.219–18.60	0.535
Round	4.56	1.09–19.24	0.039
Margin	0.13	0.019–0.885	0.037
Echogenicity	1.49	0.349–6.34	0.591
Hilar structure	1.03	0.155–6.88	0.974

In the 94 surgically treated patients, the comparison of preoperative EUS examinations to the postsurgical pathologic examination of esophageal regional lymph node metastases was analyzed first. The sensitivity of EUS in the detection of esophageal regional lymph node metastases was 80.1%, and the specificity was 55.6%. The area under the ROC curve was 0.683. There was a statistically significant difference with the area under the curve compared with 0.5 (*P* <0.05). The testing method was the z-test. EUS showed efficiency in the diagnosis of esophageal regional lymph node metastases overall.

In Table 3, we compared the RLN lymph node metastases, as determined by preoperative EUS, EBUS and CT, with post-surgical pathologic examination among patients who were treated with radical surgery.

**Table 3:** Comparison of the accuracy of RLN lymph node metastases by EUS, EBUS and CT with surgical pathology.

		Pathology		Total	sensitivity	specificity	PPV	NPV	Youden index
		+	-						
EUS	+	11	5	16	32.4%	91.7%	68.8%	70.5%	0.241
	-	23	55	78					
EBUS	+	23	15	38	67.6%	75.0%	60.5%	80.4%	0.426
	-	11	45	56					
CT	+	10	3	13	29.4%	95.0%	76.9%	70.4%	0.244
	-	24	57	81					

PPV-Positive Predictive Value, NPV-Negative Predictive Value.

The sensitivity of the diagnosis of RLN lymph node metastases by preoperative EBUS was significantly different from that by EUS (*P* = 0.004<0.05). The sensitivity of EBUS was also significantly different from that of CT (*P* = 0.007<0.05). The sensitivity of EUS and CT were not significantly different (P = 0.5>0.05).

According to the data from Table 3 and previous descriptions, we compared the sensitivity of EUS in the diagnosis of esophageal regional lymph node metastases (80.1%) and the diagnosis of para-RLN lymph node metastases (32.4%). The difference between these diagnoses was statistically significant, which indicated that EUS had a poorer sensitivity in the diagnosis of para-RLN lymph nodes compared with that of esophageal regional lymph nodes overall (pathology of lymph node = 1 (positive), χ^2^ = 21.8, *P* <0.05). Based on the data in Table 3, the sensitivity of the diagnosis of RLN lymph node metastases by preoperative EBUS showed a significant difference from the sensitivity and accuracy of diagnosis by EUS and CT. In contrast, the sensitivity of EUS and CT showed no significant difference.

To understand the effect of positions on the accuracy of the diagnosis of RLN lymph node metastases, we separately compared the accuracy of the diagnosis of right and left RLN lymph node metastases by EBUS, EBUS and CT. The results are shown in Table 4.

**Table 4:** The effect of positions on accuracy by EUS, EBUS and CT in the diagnosis of RLN lymph node metastases.

			Pathology		Total	sensitivity	specificity	accuracy
			+	-				
Right RLN lymph nodes	EUS	+	8	1	27	28.6%	98.4%	77.7%
		-	20	65	67			
	EBUS	+	18	10	28	64.3%	84.8%	78.7%
		-	10	56	66			
	CT	+	6	3	9	21.4%	95.5%	73.4%
		-	22	63	85			
Left RLN lymph nodes	EUS	+	4	3	7	30.8%	96.3%	87.2%
		-	9	78	87			
	EBUS	+	9	5	14	69.2%	93.8%	90.4%
		-	4	76	80			
	CT	+	2	5	7	15.4%	93.8%	83.0%
		-	11	76	87			

For the diagnosis of right RLN lymph node metastases, the sensitivity of EBUS was significantly different from that by EUS or CT (*P* <0.05). Additionally, no significant difference was observed in the sensitivity of this diagnosis by EUS and CT (*P* >0.05). The specificity of the diagnosis by EUS, EBUS and CT showed no significant difference (*P* >0.05). For the diagnosis of left RLN lymph node metastases, the sensitivity of EBUS showed a statistically significant difference from that by CT (*P*<0.05) but no significant difference from EUS (*P* >0.05). The sensitivity of this diagnosis by EUS and CT was not significantly different (*P* >0.05). The specificity of the diagnosis by EUS, EBUS and CT was not significantly different (*P* >0.05). The testing method was the McNemar Test. The sensitivity and accuracy of PET/CT were not significantly different from those by EUS or EBUS (*P* >0.05). The testing method was the McNemar Test.

According to the data shown in Table 4, the sensitivity of the diagnosis of right RLN lymph node metastases by EBUS was significantly different from that by EUS or CT (*P* <0.05). Additionally, no significant difference was observed in the sensitivity of this diagnosis by EUS and CT (*P* >0.05). The specificity of the diagnosis by EUS, EBUS and CT showed no significant difference (*P* >0.05). For the diagnosis of left RLN lymph node metastases, the sensitivity of EBUS showed a statistically significant difference from that of CT (*P* <0.05) but no significant difference from that of EUS (*P* >0.05). The sensitivity of this diagnosis by EUS and CT showed no significant difference (*P* >0.05). The specificity of the diagnosis by EUS, EBUS and CT showed no significant difference (*P* >0.05). The testing method was the McNemar Test.

Although positron emission tomography/computed tomography (PET/CT) is a powerful tool for predicting lymph node metastases and is considered the most sensitive means of detecting distant and regional metastasis to date, in this study, 14 of the 115 patients underwent preoperative PET/CT scans, whereas the remaining 101 patients did not undergo PET/CT examination due to the expensive cost. From our limited data, PET/CT was not superior to EBUS or EUS in the detection of RLN lymph node metastases. The result is shown in Table 5.

**Table 5:** Comparison of the accuracy of the diagnosis of right and left RLN lymph node metastasis by EBUS, EBUS and PET/CT, compared with the result from surgical pathology.

			Pathology		Total	sensitivity	specificity	accuracy
			+	-				
Right RLN lymph nodes	EUS	+	3	0	3	37.5%	100%	64.3%
		-	5	6	11			
	EBUS	+	6	1	7	75%	83.3%	78.6%
		-	2	5	7			
	PET/CT	+	5	3	8	62.5%	50%	57.1%
		-	3	3	6			
Left RLN lymph nodes	EUS	+	1	0	1	20%	100%	71.4%
		-	4	9	13			
	EBUS	+	4	0	4	80%	100%	92.9%
		-	1	9	10			
	PET/CT	+	2	1	3	40%	88.9%	71.4%
		-	3	8	11			

The sensitivity and accuracy of PET/CT showed no statistically significant difference from that by EUS or EBUS (*P*>0.05). The testing method was the McNemar Test.

According to the data from Table 5, in the diagnosis of right and left RLN lymph node metastases, the sensitivity and accuracy of PET/CT was not significantly different from that of EUS or EBUS (*P* >0.05). The testing method was the McNemar Test.

## Discussion

Patients with esophageal cancer have a high mortality rate. One of the risk factors for a poor prognosis is lymph node metastasis and recurrence. The plexus of esophageal submucosal lymphatic drainage not only penetrates laterally through the esophageal wall to drain to the adjacent lymph nodes but also contains vertical longitudinal traffic, which reveals its anatomical link to the RLN and the gastric cardia lymph nodes. Esophageal cancer can spread widely to distant lymph nodes in the early stages [9,10]. It has been shown that the RLN lymph nodes are important in the prediction of metastases to distant lymph nodes during thoracic esophageal cancer. The positivity of RLN lymph nodes for metastases indicates a wider range of lymph node metastases [3,4].

Currently, some controversy exists concerning the area of the lymph nodes that needs to be dissected in patients with thoracic esophageal cancer. Most studies have shown that three-field dissection can improve the 5-year survival rate, though others have suggested that two-field dissection is sufficient for patients with esophageal cancer [11]. Some clinical studies have revealed that the prognoses of patients with esophageal cancer with positive RLN lymph nodes and who underwent three-field dissection were significantly better than those of patients who underwent traditional two-field dissection; however, no significant difference was observed in the prognoses of patients with negative RLN lymph nodes who received either of these types of dissections. Furthermore, the three-field dissection significantly increased the incidence of RLN injury and pulmonary complications [12]. Currently, researchers in both Japan and China have agreed that RLN lymph node metastases are an important indication of extended lymphadenectomy of cervical nodes in cases of thoracic esophageal carcinoma [13,14]. An intra-operative cryosectioning or PCR test of the RLN lymph node is also commonly used to avoid unnecessary cervical lymph node dissection during esophagectomy [15]. However, these extra intra-operative tests prolong the operation time. Therefore, it is more practical to use preoperative examination for metastases in the RLN lymph node as an indication of the range of lymphadenectomy of thoracic esophageal cancer.

Currently, the determination of the stages of esophageal cancer still depends on EUS and CT scans. Our results show that EUS provides less sensitivity in the detection of metastasis of the RLN lymph node compared with that of para-esophageal regional lymph nodes according to the data presented in Table 3. This could be because the air in the trachea, bronchi, or lung tissues around the esophagus can attenuate the ultrasonic signal of EUS and reduce the visibility of metastases in the RLN lymph node, which reflects the lower sensitivity of EUS to detect RLN lymph node metastases.

It is difficult to detect and diagnose lymph node metastases of less than 1 centimeter in diameter by a conventional CT scan. Based on our data, we found no statistically significant difference in the sensitivity of the diagnosis of RLN lymph node metastases by CT scan and EUS (Table 3).

According to our results, EBUS demonstrated a better sensitivity than EUS or CT scan for the diagnosis of RLN lymph node metastases. The differences were statistically significant (Table 3). Through contact with the water-filled balloon and detection from the tracheal side, EBUS maximally avoids any interference from air in the trachea. Para-tracheal lymph nodes smaller than 5 millimeters in diameter are more visible by EBUS. This superior sensitivity showed some differences according to the positions of both right and left RLN lymph nodes (Table 4). The sensitivity of the diagnosis of right RLN lymph node metastases by EBUS was significantly different from that by EUS or CT. To detect left RLN lymph node metastases, EBUS was significantly different from CT but not from EUS. The left RLN passes through a longer distance than the right RLN. The common visual field of EUS and EBUS to detect lymph nodes is also longer along the left RLN, which caused the similar sensitivities of EBUS and EUS on the left side. However, the visual field of EBUS along the right RLN may give EBUS more chances to detect metastatic lymph nodes than that of EUS, which caused the superior sensitivity of EBUS on the right side.

Although PET/CT is considered to be the most sensitive means of detecting distant and regional metastases and a powerful tool to diagnose lymph node metastases, the limitation of our study is that only 14 of the 115 patients underwent a preoperative PET/CT scan; the remaining 101 patients did not undergo PET/CT examination due to the expensive cost. From the limited results (Table 5), PET/CT was not superior to EBUS or EUS to detect RLN lymph node metastases, including the right and left sides. Considering the cost-benefit ratio, comparison of preoperative EBUS and PET/CT to detect RLN lymph node metastases requires further research.

In addition, 14 patients who were recruited but were not included in the study cohort were diagnosed with tracheal cancer invasions by EBUS in this study. Nine of these 14 patients failed to be assessed by EUS due to the presence of obstructive esophageal tumors. Another 7 patients were diagnosed with tracheal invasions by esophageal cancer by both EUS and EBUS. All 21 patients were treated with radiotherapy and chemotherapy instead of radical surgery. In this study, we could not directly prove the real tracheal invasion by performing surgeries in the case of patients who had a highly suspicious tracheal invasion by EBUS because the thoracic surgeons preferred to avoid an invalid exploratory thoracotomy that would likely not benefit the patient. As circumstantial evidence, all 94 patients who were diagnosed as negative for a tracheobronchial tree invasion by EBUS demonstrated no invasion of the trachea, as observed during radical surgery. Because of the interference by air and the narrow space between the trachea and the esophagus, EUS has less sensitivity in the diagnosis of esophageal cancer that has invaded the tracheobronchial tree [16]. Our observations indicate that EBUS may be an additional complement in the preoperative exclusion of patients with tracheal invasion, especially in the case of esophageal stricture.

Endobronchial ultrasound-guided transbronchial needle aspiration (EBUS-TBNA) has shown a promising ability in the diagnosis of lymph node metastasis. In this study, three patients who were strongly suspected to have RLN lymph node metastases underwent EBUS-TBNA, and the results showed positive metastases in all three patients. However, as for thoracic surgeons, EBUS-TBNA for recurrent laryngeal lymph nodes may cause post-procedural hematomas around the lymph node that can interfere with lymph node dissection in a clean plane, which will increase the risk of RLN paralysis. In addition, TBNA is good for the diagnosis of positive lymph node metastases, but it is still difficult to exclude false negative cases. TBNA also has the risks of trauma and complications. Therefore, thoracic surgeons prefer to use non-invasive methods to evaluate lymph node metastases preoperatively, and we tried to predict the RLN lymph node metastases based on morphologic features in this study. Although the thoracic surgeons did not make clinical decisions based on EBUS imaging alone, they indeed raised the possibility of a greater range of preoperative lymph node dissections if the results of EBUS indicated RLN lymph node metastases. The application of EUS-TBNA in the diagnosis of RLN lymph node metastases may be suitable for nonsurgical treatment.

According to the results shown in Table 2, a round shape and distinct margins were the influential morphologic predictors of RLN lymph node metastases. Recently, the use of elastic scattering spectroscopy non-invasive methods to evaluate sentinel lymph node metastases in breast cancer has been reported [17]. This technique might provide more criteria for morphologic analysis and could be a candidate for an EBUS non-invasive diagnosis of lymph node metastases in esophageal cancer.

In conclusion, although clinical decisions based on EBUS imaging still requires further research, EBUS can enhance the sensitivity of preoperative diagnosis of RLN lymph node metastases in patients with thoracic esophageal cancer and may indicate tracheal invasion in the case of esophageal stricture; thus, it can serve as a useful complementary examination to conventional preoperative EUS and CT examinations. A combination of EBUS and EUS in the diagnosis of esophageal cancer can provide a more precise determination of preoperative staging of esophageal cancer and may provide better information for the scope of the lymphadenectomy. This combination will help further develop suitable preoperative individualized treatment plans.

## References

[1] Pfau PR, Perlman SB, Stanko P, Frick TJ, Gopal DV, Said A, et al The role and clinical value of EUS in a multimodality esophageal carcinoma staging program with CT and positron emission tomography. Gastrointest Endosc. 2007; 65:377–384. . 17321235 10.1016/j.gie.2006.12.015

[2] Tan Z, Ma G, Zhao J, Bella AE, Rong T, Fu J, et al Impact of thoracic recurrent laryngeal node dissection: 508 patients with tri-incisional esophagectomy. J Gastrointest Surg. 2014;18:187–193. 10.1007/s11605-013-2411-2 24241966

[3] Taniyama Y, Nakamura T, Mitamura A, Teshima J, Katsura K, Abe S, et al A strategy for supraclavicular lymph node dissection using recurrent laryngeal nerve lymph node status in thoracic esophageal squamous cell carcinoma. Ann Thorac Surg. 2013; 95:1930–1937. 10.1016/j.athoracsur.2013.03.069 23642437

[4] Shimada H, Okazumi S, Shiratori T, Akutsu Y, Matsubara H. Mode of lymphadenectomy and surgical outcome of upper thoracic esophageal squamous cell carcinoma. J Gastrointest Surg. 2009; 13:619–625. 10.1007/s11605-008-0790-6 19156473

[5] Ohnishi R, Yasuda I, Kato T, Tanaka T, Kaneko Y, Suzuki T, et al Combined endobronchial and endoscopic ultrasound-guided fine needle aspiration for mediastinal nodal staging of lung cancer. Endoscopy. 2011; 43:1082–1089. 10.1055/s-0030-1256766 21971924

[6] Tamiya M, Okamoto N, Sasada S, Shiroyama T, Morishita N, Suzuki H, et al Diagnostic yield of combined bronchoscopy and endobronchial ultrasonography, under LungPoint guidance for small peripheral pulmonary lesions. Respirology. 2013; 18:834–839. 10.1111/resp.12095 23586738

[7] Faige DO. EUS in patients with benign and malignant lymphadenopathy. Gastrointest Endosc. 2001; 53:593–598. . 11323584 10.1067/mge.2001.114060

[8] Shimizu S, Hosokawa M, Itoh K, Fujita M, Takahashi H, Shirato H, et al Can hybrid FDG-PET/CT detect subclinical lymph node metastasis of esophageal cancer appropriately and contribute to radiation treatment planning? A comparison of image-based and pathological findings. Int J Clin Oncol. 2009; 14:421–425. 10.1007/s10147-009-0893-4 19856050

[9] Kuge K, Murakami G, Mizobuchi S, Hata Y, Aikou T, Sasaguri S. Submucosal territory of the direct lymphatic drainage system to the thoracic duct in the human esophagus. J Thorac Cardiovasc Surg. 2003 125:1343–1349. . 12830054 10.1016/s0022-5223(03)00036-9

[10] Mizutani M, Murakami G, Nawata S, Hitrai I, Kimura W. Anatomy of right recurrent nerve node: why does early metastasis of esophageal cancer occur in it? Surg Radiol Anat. 2006; 28:333–338. . 16718401 10.1007/s00276-006-0115-y

[11] Law S, Wong J. Two-field dissection is enough for esophageal cancer. Dis Esophagus. 2001; 14:98–103. . 11553217 10.1046/j.1442-2050.2001.00164.x

[12] Shiozaki H, Yano M, Tsujinaka T, Inoue M, Tamura S, Doki Y, et al Lymph node metastasis along the recurrent nerve chain is an indication for cervical lymph node dissection in thoracic esophageal cancer. Dis Esophagus. 2001;14(3–4):191–196. . 11869318 10.1046/j.1442-2050.2001.00206.x

[13] Takemura M, Osugi H, Takada N, Kinoshita H, Higashino M. Prognostic factors in patients with squamous oesophageal cancer associated with solitary lymph node metastasis after oesophagectomy and extended lymphadenectomy. Oncol Rep. 2003; 10:75–80. . 12469148

[14] Li H, Yang S, Zhang Y, Xiang J, Chen H. Thoracic recurrent laryngeal lymph node metastasis predict cervical node metastasis and benefit from three-field dissection in selected patients with thoracic esophageal squamous cell carcinoma. J Surg Oncol. 2012; 105:548–552. 10.1002/jso.22148 22105736

[15] Miyata M, Yano M, Doki Y, Yasuda T, Yoshioka S, Sugita Y, et al A prospective trial for avoiding cervical lymph node dissection for thoracic esophageal cancers, based on intra-operative genetic diagnosis of micrometastasis in recurrent laryngeal nerve chain nodes. J Surg Oncol. 2006; 93:437–438. . 16615150 10.1002/jso.20453

[16] Garrido T, Maluf-Filho F, Sallum RA, Figueiredo VR, Jacomelli M, Tedde M. Endobronchial ultrasound application for diagnosis of tracheobronchial tree invasion by esophageal cancer. Clinics. 2009; 64:499–504. . 19578652 10.1590/S1807-59322009000600003 PMC2705145

[17] Austwick MR, Clark B, Mosse CA, Johnson K, Chicken DW, Somasundaram SK, et al Scanning elastic scattering spectroscopy detects metastatic breast cancer in sentinel lymph nodes. J Biomed Opt. 2010; 15:047001 10.1117/1.3463005 20799832 PMC2917446

